# Complex Diffractive Optical Elements Stored in Photopolymers

**DOI:** 10.3390/polym11121920

**Published:** 2019-11-21

**Authors:** Roberto Fernández, Sergi Gallego, Andrés Márquez, Cristian Neipp, Eva María Calzado, Jorge Francés, Marta Morales-Vidal, Augusto Beléndez

**Affiliations:** 1 Instituto Universitario de Física Aplicada a las Ciencias y las Tecnologías, Universidad de Alicante, P.O. Box 99, 03080 Alicante, Spain; andres.marquez@ua.es (A.M.); cristian@ua.es (C.N.); evace@ua.es (E.M.C.); jfmonllor@ua.es (J.F.); marta.morales@ua.es (M.M.-V.); a.belendez@gcloud.ua.es (A.B.); 2 Departamento de Física, Ingeniería de Sistemas y Teoría de la Señal, Universidad de Alicante, P.O. Box 99, E03080 Alicante, Spain

**Keywords:** holographic recording materials, photopolymers, diffractive elements, optical axicons

## Abstract

We study the recording of complex diffractive elements, such as achromatic lenses, fork gratings or axicons. Using a 3-D diffusion model, previously validated, we are able to predict the behavior of photopolymer during recording. The experimental recording of these complex elements is possible thanks to a new generation spatial light modulator capable of generating periodic and aperiodic profiles. Both experimental and theoretical are analyzed and compared. The results show not only the good response of theoretical model to predict the behavior of the materials, but also the viability of photopolymers to store these kind of elements.

## 1. Introduction

The advantages of photopolymers as recording media for holographic applications and fabrication of photonic devices, such as waveguides, diffractive optical elements (DOEs) or 2-dimensional photonic structures have been widely discussed [1,2,3]. One of the most used materials for these applications is the one based on polyvinyl alcohol/acrylamide (PVA/AA). This material showed outstanding characteristics [4,5,6] and interesting results were obtained in our firsts approaches to complex DOEs recording such as blazed gratings [7] or diffractive lenses [8]. Thanks to the addition of an index matching system [5,6], the shrinkage effects of the surface were minimized. Thus, the phase depth of 2π needed to record complex DOEs was achieved in this family of photopolymers.

In the diffractive lenses, previously studied, a change in wavelength modifies the focal length and, therefore, the diffraction efficiency (DE) is affected. Although high quality point spread function (PSF) was obtained for particular wavelength. Consequently, these elements are intended only for applications with monochromatic light sources. On this basis, it is interesting to count on achromatic lenses, or achromats, which shows the same behaviour for different wavelengths. In diffractive optics, these can be obtained by the combination of two or more diffractive lenses [9]. Thanks to DOE’s advantages and its capability of performing different functions simultaneously, it is possible to multiplex various lenses in the same DOE by using different multiplexing schemes [10,11,12]. The versatility of fabricating DOEs using a new generation spatial light modulator (SLM) allowed us to test different spatial multiplexing schemes in a simple way. A wavefront can be modulated without moving any physical parts on the setup and obtain patterns unable to be obtained by traditional interference holography.

The possibilities of using a SLM to generate DOEs go further and allowed us to study the viability of recording different types of complex DOEs in photopolymer. A very interesting DOE are the fork gratings. These are one of the easiest ways to create vortex singularities, also called optical vortices [1,13,14,15] which attracted much attention due to their possible applications in trapping and manipulation of particles [16,17], atom trapping and guiding [18], quantum optics [19], interferometry [20] or optical and free-space communications [21] among others. Optical vortices are commonly characterized by a phase singularity and a helical phase front around them. The phase of the central point of the helix is undefined and therefore the intensity is zero, resulting in the characteristic toroidal intensity profile.

Another very interesting DOE are diffractive axicons. This concept was first used by J.H. McLeod [22] and defined as an optical system which images a point source on its axis of revolution to a range of points along its axis. This element generates a narrow focal line along the optical axis and it is used to generate non-diffracting Bessel beams. These have an extended depth of focus when compared with Gaussian Beams [23] and importance in applications such as optical coherence microscopy, atom guiding or micro-drilling [24,25,26].

In this paper, we evaluate the recording of achromats, fork gratings and diffractive axicons in photopolymers. Using a three dimensional diffusion model, previously validated by [6,8,27], we predicted the behaviour of the material during DOE’s recording. On the other hand, a SLM-based setup was used to experimentally record these DOEs on the material. We compare both theoretical and experimental data and justify the suitability of material for these applications.

## 2. Diffusion Model

For DOEs which present symmetry, such as for gratings or axicons, the three dimensional behaviour during recording can be described by two spatial dimension functions. We used the following general equations for light propagating in Z positive direction:

(1) $$\begin{array}{cc} \; & \frac{\partial \phi^{\left(m\right)}\left(x,z,t\right)}{\partial t}=\frac{\partial }{\partial x}D_{m}\left(t\right)\frac{\partial \phi^{\left(m\right)}\left(x,z,t\right)}{\partial x}+\frac{\partial }{\partial z}D_{m}\left(t\right)\frac{\partial \phi^{\left(m\right)}\left(x,z,t\right)}{\partial z}\\ \; & -F_{R}\left(x,z,t\right)\phi^{\left(m\right)}\left(x,z,t\right) \end{array}$$

(2) $$\frac{\partial \phi^{\left(p\right)}\left(x,z,t\right)}{\partial t}=F_{R}\left(x,z,t\right)\phi^{\left(m\right)}\left(x,z,t\right)$$

where $D_{m}$ is the diffusion of monomer inside the material, $F_{R}$ represents the polymerization rate and $\phi^{\left(m\right)}$ and $\phi^{\left(p\right)}$ are the monomer and polymer volume fractions respectively. The initial value for $D_{m}$ was measured through diffractive techniques following the procedure of [6]. For elements without symmetry, i.e., spherical lenses, it is necessary to add another dimension, related to *y*variable. Then, Equation (1) remains as follows:

(3) $$\begin{array}{cc} \; & \frac{\partial \phi^{\left(m\right)}\left(x,y,z,t\right)}{\partial t}=\frac{\partial }{\partial x}D_{m}\left(t\right)\frac{\partial \phi^{\left(m\right)}\left(x,y,z,t\right)}{\partial x}+\frac{\partial }{\partial y}D_{m}\left(t\right)\frac{\partial \phi^{\left(m\right)}\left(x,z,t\right)}{\partial y}\\ \; & +\frac{\partial }{\partial z}D_{m}\left(t\right)\frac{\partial \phi^{\left(m\right)}\left(x,y,z,t\right)}{\partial z}-F_{R}\left(x,z,t\right)\phi^{\left(m\right)}\left(x,z,t\right) \end{array}$$

(4) $$\frac{\partial \phi^{\left(p\right)}\left(x,y,z,t\right)}{\partial t}=F_{R}\left(x,y,z,t\right)\phi^{\left(m\right)}\left(x,y,z,t\right)$$

$F_{r}$ depends on the recording intensity and chemical kinetics. This dependence can be described by the following equation:

(5) $$F_{R}\left(x,y,z,t\right)=k_{R}\left(x,y,z,t\right)I{\left(x,y,z,t\right)}^{\gamma }=k_{R}\left(x,y,z,t\right)I{\left(x,y\right)}^{\gamma }e^{-\alpha (t)\gamma }$$

where $k_{r}$ stands for the polymerization constant, *I* corresponds to the recording intensity, γ represents the relationship between intensity and polymerization rate and α denotes the coefficient of light attenuation.

The monomer and polymer’s volume fraction are given by $\phi_{i}=x_{i}v_{i}/\sum x_{i}v_{i}$, where $x_{i}$ and $v_{i}$ are the molar fraction and the molar volume of the *i*th component respectively. Using the Lorentz–Lorenz equation, the refractive index, *n*, of the layer as a function of the volume fraction variations of each component can be calculated as follows [28,29]:

(6) $$\frac{n^{2}-1}{n^{2}+2}=\frac{n_{m}^{2}-1}{n_{m}^{2}+2}\phi^{\left(m\right)}+\frac{n_{p}^{2}-1}{n_{p}^{2}+2}\phi^{\left(p\right)}+\frac{n_{b}^{2}-1}{n_{b}^{2}+2}\left(1-\phi^{\left(m_{0}\right)}\right)$$

where the average initial value for the monomer volume fraction is represented by $\phi^{\left(m_{0}\right)}$ and $n_{m}$, $n_{p}$ and $n_{b}$ are the monomer, polymer and support matrix refractive index, respectively.

## 3. Experimental Setup

Experimental setup was based on a new generation SLM (model PLUTO, Holoeye, Berlin, Germany), equipped with a Liquid crystal on silicon(LCoS) microdisplay with a resolution of 1920 × 1080 pixels and a pixel pitch of 8 μm. This device was previously characterized by our research group [30].

**Figure 1:**
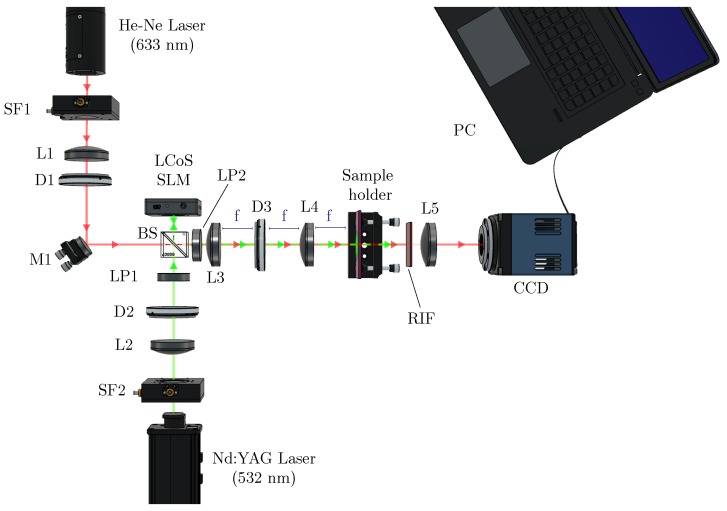
Experimental setup. BS: beam splitter, Mi: mirror, SFi: spatial filter, LP: lineal polarizer, Li: lens, Di: diaphragm, RIF: red interference filter and PC: data recorder.

All the DOEs used in this work were generated by computer using MATLAB (Mathworks Natick, MA, USA) software and introduced in the SLM to be projected in the material as an intensity distribution. We measured the formation of the DOE in real time using the setup shown in Figure 1. The SLM was located in the recording arm of the setup, between two polarizers (LPs) to produce amplitude modulation. The recording beam was formed by a solid-state laser (model Verdi Nd:YVO${}_{4}$, Coherent, CA, USA), operating at a wavelength of 532 nm (green light). At this wavelength, the dye used in the material’s composition, yellowish eosine, presents maximum absorption. To have direct access to Fourier plane, a 4f system was used. Using this system, we imaged the intensity distribution onto the recording material. L3 and L4 were two identical lenses with a common focal point. Between these two lenses, there was a diaphragm (D3) used to eliminate the pixilation of the LCD screen of the SLM. DEs were measured in real time as:

(7) $$DE=\frac{I_{i}}{I_{I}}$$

where $I_{i}$ and $I_{I}$ represent the intensity of diffraction orders and the incident intensity, respectively. The analyzing beam, used to observe in real time the changes produced during photopolymerization process, was formed by a s-polarized He-Ne (633 nm) laser. At this wavelength the material does not present light absorption. A lens (L1) was placed to collimate the beam before reaching the material. The aperture of this beam was controlled by a diaphragm (D1). A non-polarizing beamsplitter (BS) made both beams follow the same path. To cut the recording beam after passing through the material, a red interference filter (RIF) was used. Thus, we ensured that only the information of the analyzing beam was recorded on the high dynamic range CCD camera (model pco.1600, pco.imaging) placed at the end of the setup. The camera had a resolution of 1600 × 1200 and a pixel size of 7.4 μm × 7.4 μm. Located before the CCD camera, there was a lens (L5) used to separate the diffraction behind material and obtain the Fraunhofer diffraction pattern. In lenses recording experiments this lens was removed as the element itself was the lens. The different intensity patterns were also evaluated placing the CCD camera in the material’s plane to record the pattern imaged by SLM plane. The recording intensity used was 0.5 mW/cm${}^{2}$.

The material used in this work was a photopolymer based on polyvinyl alcohol/acrylamide (PVA/AA). Its particular concentrations are shown in Table 1. It was composed of AA as the polymerizable monomer, PVA as the binder, *N*,*N*${}^{\prime }$-methylene-bis-acrylamide (BMA) as the crosslinking monomer, triethanolamine (TEA) as the coinitiator, and plasticizer and yellowish eosin (YE) as the dye.

The layer was prepared by depositing 30 mL of preparation by force of gravity on a glass substrate (25 cm × 25 cm) and left in the dark for about 36 h (*RH* = 40–45%, *T* = 20–23${}^{\circ }$). After this time, part of the water was evaporated and the layer had enough mechanical resistance to be cut without deformation. The final solid layer had a thickness of 90 ± 5 μm. For thickness measurement, firstly we used an ultrasound display (Neurtek). Then, to confirm the value measured, we recorded a holographic grating to fitting the angular response around Bragg’s angle [31].

## 4. Results and Discussion

### 4.1. Fork Grating and Diffractive Axicon

**Figure 2:**
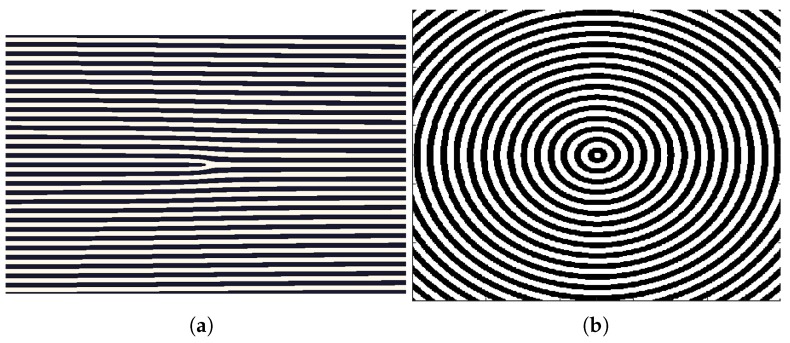
Intensity distributions of (**a**) fork grating and (**b**) diffractive axicon.Intensity distributions

The different intensity patterns were introduced in the SLM, working in amplitude mode, as mentioned in the previous section. In Figure 2a,b the intensity patterns of fork grating and diffractive axicon, respectively, are shown. Fork grating consists of a sinusoidal or blazed grating with a fork-shaped division in its center. The number of tines of the central fork determine the topological charge (*L*). This parameter is defined as the number of 2π phase changes along the azimuthal direction within a distance of λ. *L* parameter affects to the size of the produced toroidal beam, which increases as *L* becomes larger. In Figure 2b, a diffractive axicon is shown, previously defined as an element which form a focal line along optical axis, instead of focusing it in a point. Axicons are used to generate Bessel beams, or non-diffracting beams with the characteristic ring-shaped form or to image a point source into a line along the optical axis, increasing the depth of focus.

Using the diffusion model, we simulated the recording of a 80 μm fork grating at the central point in Fraunhofer domain. These results were compared with the experimental recording of the same fork grating.

**Figure 3:**
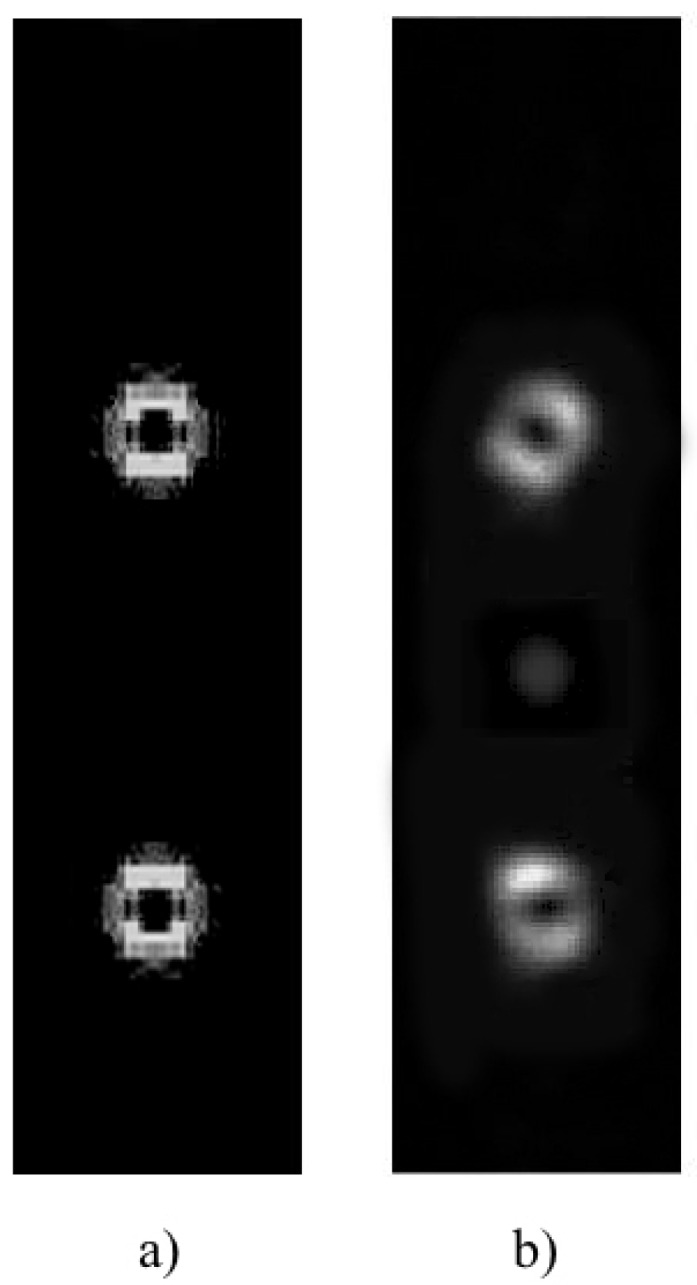
Far-field diffraction patterns of fork grating. (**a**) Simulated diffraction pattern; (**b**) experimental diffraction pattern recorded by CCD camera.

**Figure 4:**
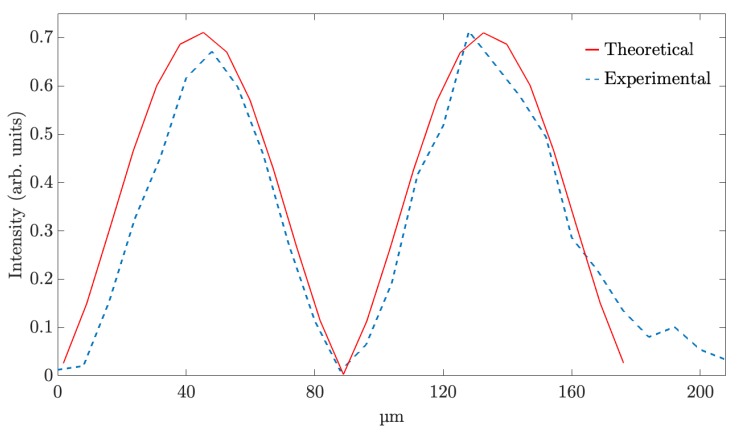
Intensity profile of theoretical and experimental fork gratings.

Figure 3a,b shows the simulated and experimental diffraction pattern of the fork grating. The characteristic toroidal shape can be seen together with the agreement between the experimental and the predicted results by the diffusion model. In the experimental diffraction pattern recorded by CCD camera, a weak zero order, with a mean intensity lower than the 25% of the mean intensity of the ±1 orders, is still present. This is produced due to the low pass filtering introduced by the experimental setup, which affects the shape of the profile, causing a deviation of the theoretical profile. Nevertheless, in Figure 4, a good agreement between both experimental (dashed line) and theoretical (continuous line) intensity profiles can be seen, showing the characteristic intensity peaks with zero intensity value between them. The experimental profile was extracted from a vertical line of the image recorded by the CCD camera and the theoretical one was generated using the diffusion model.

**Figure 5:**
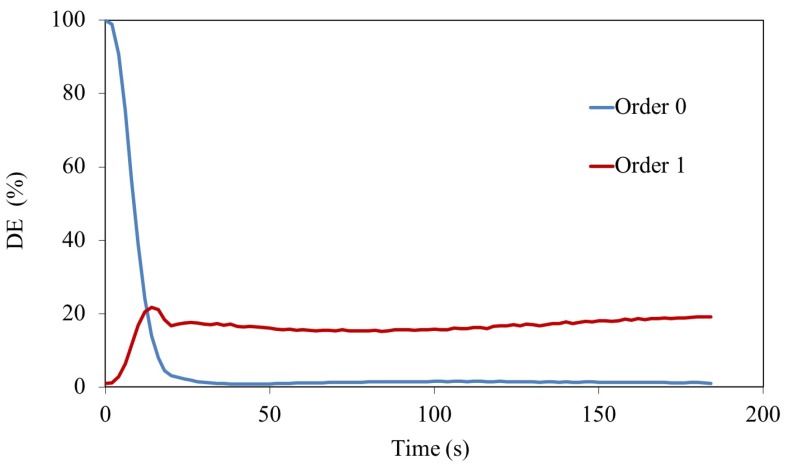
DE of the Fork grating recorded in 90 μm AA/PVA material.

In Figure 5, the DE measured for the first order of the experimental fork grating can be observed. The maximum value of DE for the first order was over 20%, a value reached after 20 s of exposure time in a 90 μm thickness layer. This value is higher than the one obtained for fork grating recording in different systems [1,32] despite it is affected by low pass filtering introduced by setup.

**Figure 6:**
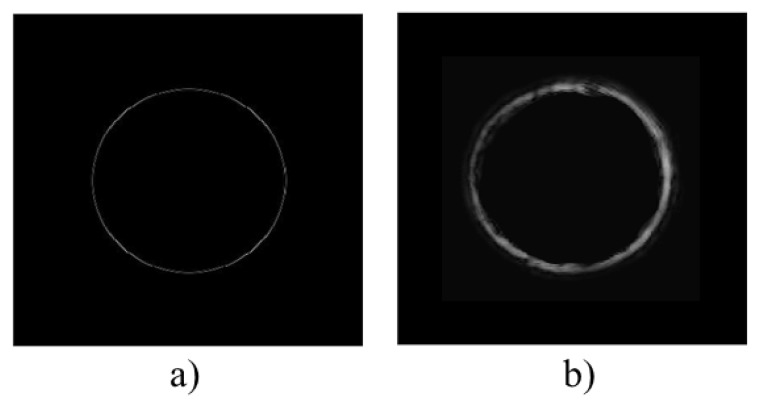
Far-field diffraction patterns of diffractive axicon. (**a**) Simulated diffraction pattern; (**b**) experimental diffraction pattern recorded by CCD camera.

**Figure 7:**
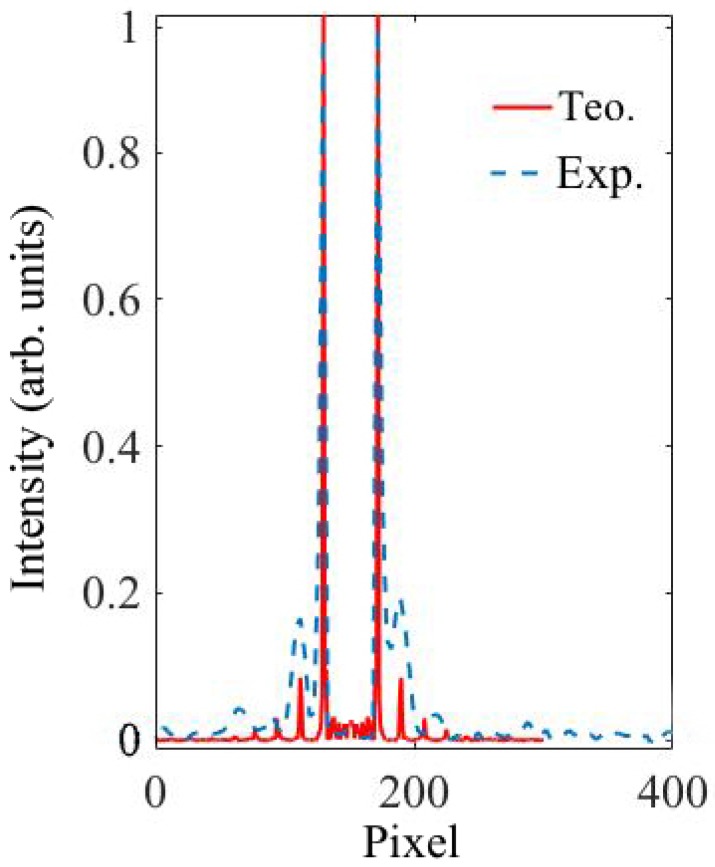
Comparison of intensity measured along a horizontal line of experimental diffraction pattern of diffractive axicon and theoretical intensity obtained through diffraction model.

The far field diffraction pattern of a diffractive axicon was also simulated and compared to the experimental diffraction pattern. Figure 6 shows the comparison between theoretical and experimental far field diffraction pattern. The recorded axicon corresponded to the prediction made by the model, showing the good behaviour of PVA/AA material, regardless of the recorded profile. The intensity along a vertical line was measured for experimental data and compared to theoretical intensity distribution for an axicon with same characteristics of the experimental one. Figure 7 shows this comparison and the good agreement between experimental and theoretical data.

**Figure 8:**
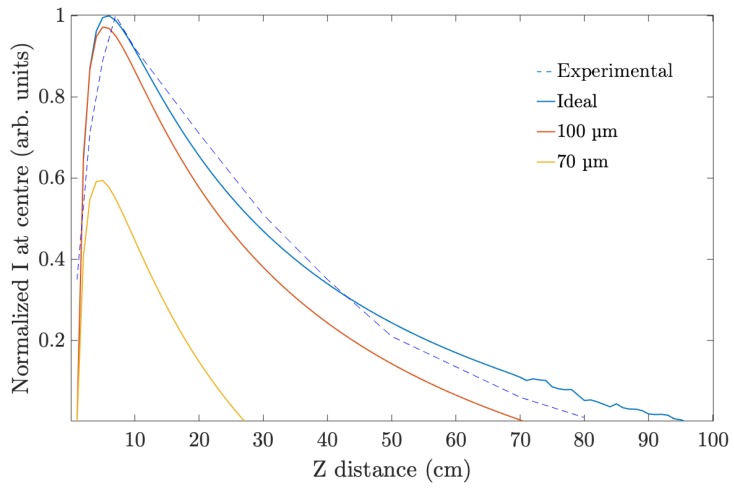
Normalized intensity at the center of a simulated axicon as a function of the distance for different material: an ideal material; a 100 μm thickness material and a 70 μm thickness material.

Fresnel equations were used to simulate intensity at the center of the axicon as a function of the distance in PVA/AA and to compare the results with the obtained through the experimental setup. Figure 8 shows this intensity for an axicon recorded in a 90 μm PVA/AA photopolymer layer. In the same figure, the simulation of the recording of an axicon in PVA/AA material of two different thickness, 70 and 100 μm compared to the simulation of an axicon recorded into an ideal material is shown, with the parameters optimized to reach a two pi phase depth and ideal binary illumination. The data were normalized by the maximum intensity reached for the ideal case. The influence of the sample’s thickness can be clearly seen. Although in all the cases the behavior with the distance was similar, the maximum intensity reached in each case was different. The 100 μm material exhibited a behavior similar to the ideal axicon, probing the great capacities and good response of the used material. For the thinnest layer, the maximum intensity reached was around 50% lower when compared to the ideal case. With lower thickness, during the recording process, the phase depth modulation does not achieve its ideal 2π value. Therefore, the maximum DE achievable is lower than the one reached by an element recorded in a material with optimal thickness. This resulted in a lower performance of the axicon recorded on the thin material.

### 4.2. Achromatic Lenses

**Figure 9:**
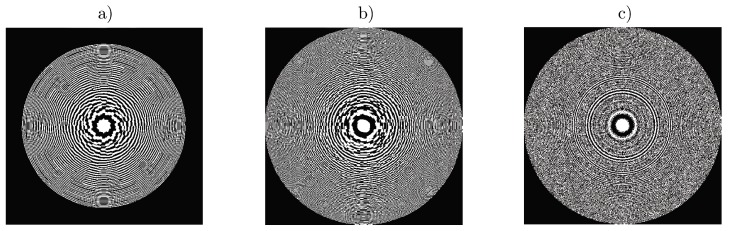
Achromatic lenses multiplexing schemes: (**a**) 30 sectors in three zones; (**b**) random multiplexing 8 × 8 pixels and (**c**) random multiplexing 1 × 1 pixels.

Figure 9 shows three different schemes of spatial multiplexing used to design apochromatic lenses, previously developed in [12]. First scheme used, shown in Figure 9a, consists of an annular sector multiplexing of three different lenses (channels) designed for 633, 514 and 458 nm, i.e., for red, green and blue wavelengths. The aperture of the lens was divided into three different rings and thirty sectors. In the first ring, channels are distributed in an ordered sequence of pixels, where each sector is filled with the pixels of red, green and blue channels, following this order until completing the ring. In the second ring and third ring, this order is switched, starting by green channel in second ring, followed by blue and green and starting by blue channel in third ring, followed by red and green channels.

The second multiplexing scheme is shown in Figure 9b,c. In this case, a random multiplexing technique was used. Whole aperture was divided in subapertures randomly distributed among the three channels. We used two different subaperture sizes, 8 × 8 and 1 × 1 pixels (Figure 9b,c, respectively). The probability was the same for each channel, but it is also possible to change the weight of each channel, changing their probabilities.

**Figure 10:**
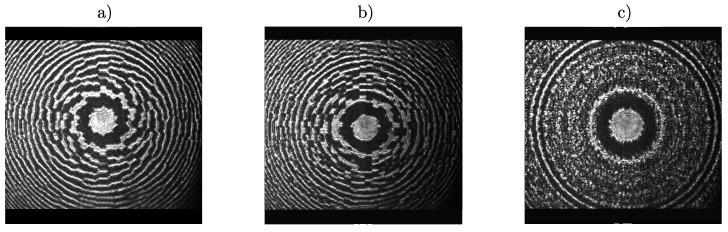
Image of the experimental achromatic lenses recorded in material’s plane using CCD camera: (**a**) 30 sectors in three zones; (**b**) random multiplexing 8 × 8 pixels and (**c**) random multiplexing 1 × 1 pixels.

The three different schemes were loaded into the LCoS SLM and their experimental images, recorded by placing the CCD camera in the material plane, are shown in Figure 10. As we previously discussed in [8,33], to develop large diameter lenses it is necessary to take into account that focal length and pixel size are factors which determine the maximum radius of the lenses. Otherwise, crosstalk effects between consecutive pixels, which can affect ring shape, and an increasing in the in the focal length of the recorded lens, may occur.

**Figure 11:**
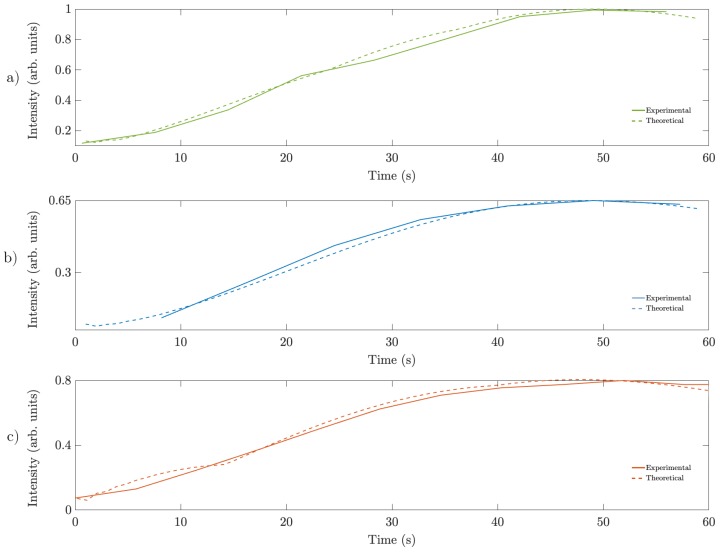
Simulated normalized intensity as a function of recording time at the focal point for different multiplexing schemes. (**a**) 30 sectors; (**b**) random multiplexing 8 × 8 and (**c**) random multiplexing 1 × 1.

**Figure 12:**
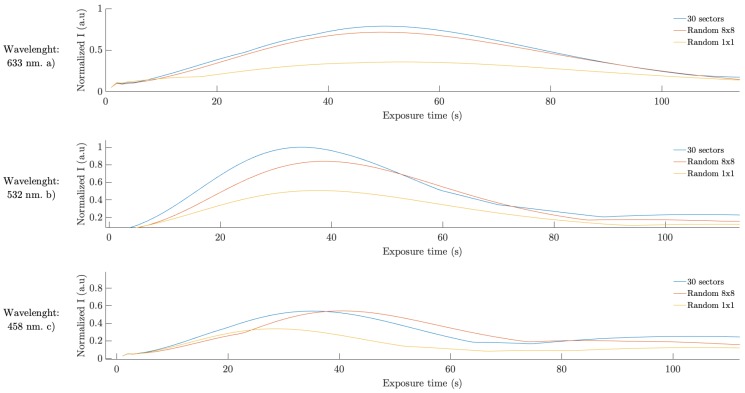
Simulated normalized intensity as a function of recording time at the focal point for different wavelength illumination. (**a**) 633 nm; (**b**) 532 nm and (**c**) 458 nm.

To achieve a realistic simulation of the lenses formation in the photopolymer, we introduced the real intensity distributions, captured in the material plane by CCD camera, into the model. Simulated normalized intensity as a function of recording time at the focal point for different multiplexing schemes is shown in Figure 11, together with the experimental results for each scheme. All the results were normalized by the maximum intensity of scheme (a), the highest intensity value reached. All schemes presents similar focusing time but they reach different maximum intensities. Scheme (b) has the lowest maximum intensity, while scheme (c) reaches near 80% of the maximum intensity reached by scheme (a). It is worth noting the good agreement between the theoretical results and the experimental ones. We also used the diffusion model to evaluate the efficiency of the different schemes for different wavelength illuminations. Figure 12 shows the behavior of these schemes for 633, 532 and 458 nm. The results shown were normalized by the maximum value obtained, which corresponds to scheme (a) for 532 nm wavelength illumination. The maximum intensity values were measured for scheme (a), followed by scheme (b). For 633 nm illumination, the focusing time is similar for the three schemes, around 50 s. Differences in the focusing time are present for 532 and 458 nm illumination. For 532 nm, the maximum intensity is reached at 35 s of exposure for scheme (a), after 39 s for scheme (b) and after 38 s for scheme (c). For 458 nm, the maximum intensity is reached at 35, 41 and 29 s for schemes (a), (b) and (c) respectively.

### 4.3. Helical Axicon

**Figure 13:**
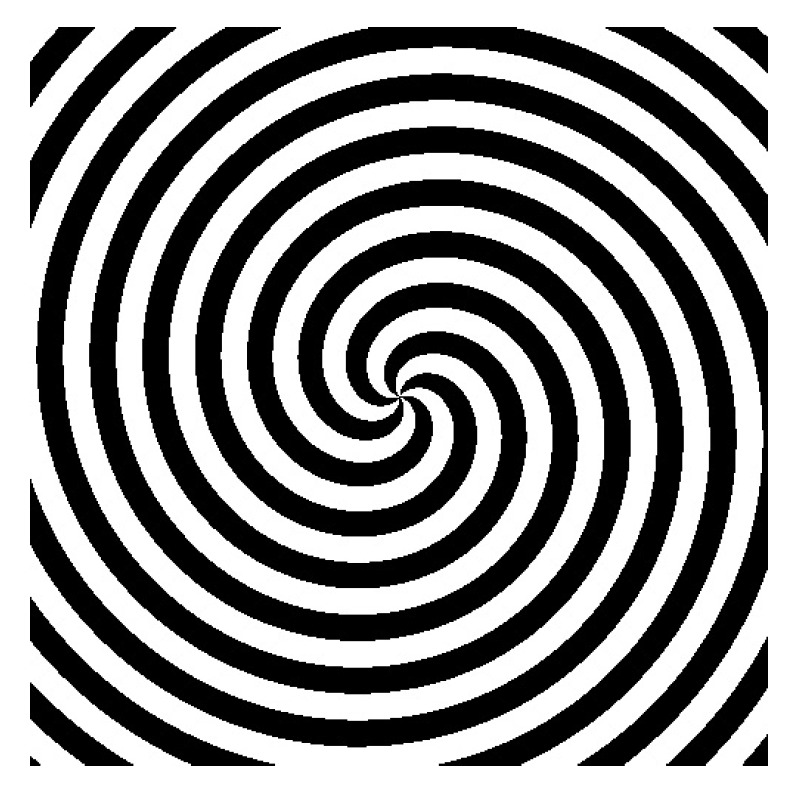
Helicoidal axicon intensity profile to be loaded in the LCoS SLM.

**Figure 14:**
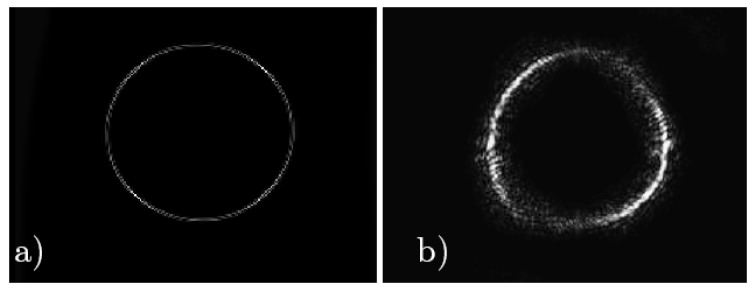
Far field diffraction pattern of helicoidal axicon. (**a**) Theoretical; (**b**) experimental.

**Figure 15:**
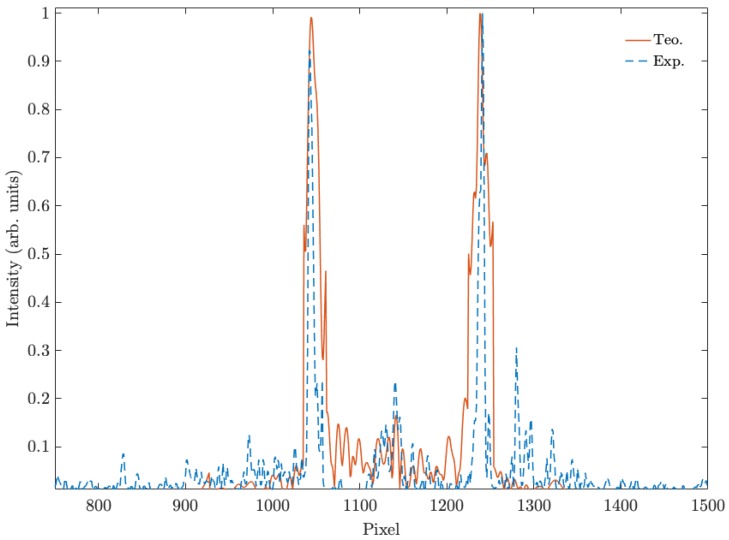
Comparison of intensity measured along a horizontal line of experimental diffraction pattern of helicoidal axicon and theoretical intensity obtained through diffraction model.

Fork gratings and diffractive axicons are used to generate Bessel beams characterized by a dark central core which propagates in free space without any spreading due to diffraction. Recently, optical beams that rotate around the optical axis are becoming an important focus of attention [34,35,36]. Helical axicons are hybrid DOEs formed by the combination of an axicon and a spiral phase plate (SPP). The latter is used to generate ring-patterned beams with helical wavefronts. Thus, by using a composite element that has the functions of both the spiral phase plate and axicon, a High-Order Bessel Beam can be generated in just one pass. In Figure 13, the intensity pattern of the helicoidal axicon, to be loaded in LCoS SLM, is shown. Figure 14 shows the comparison between the theoretical and experimental phase profile of a helical axicon (tilt angle $\theta =1^{\circ }$ and topological charges L=3). The model was able to predict the recording of this complex element in PVA/AA material, which showed its great versatility also working with this DOE. Figure 15 shows the intensity along a vertical line measured from experimental data and the theoretical intensity obtained using the diffusion model. The helicoidal axicon was simulated using the same characteristics of the experimental one. As for the diffractive axicon, there is a good agreement between experimental and theoretical results regardless of the DOE being simulated.

## 5. Conclusions

In this paper, we evaluated the suitability of our diffusion model to simulate the recording of different complex DOEs, such as fork gratings, diffractive axicons, achromatic lenses, and helicoidal axicons in a PVA/AA-based photopolymer. To validate the results obtained through the model, we recorded experimentally these complex DOEs thanks to a setup based on a LCoS SLM. Modulating the wavefront without the need for moving any physical parts on the setup allowed us to easily test multiple DOE schemes. The results obtained showed the capacities of the diffusion model to predict the behavior of the material during the recording of these complex DOEs. Moreover, for the achromatic lenses, the model allowed us to evaluate the influence of factors such as the exposure time or the recording wavelength for a particular material. This is particularly relevant as the selection of the material is one of the key aspects to obtain the desired results. In this sense, it is worth noting also the good response of the PVA/AA photopolymer storing the complex schemes evaluated, similar to the one exhibited for simpler DOEs. 

## Figures and Tables

**Table 1:** Composition of liquid solution for PVA/AA-based photopolymer.

Tea (mL)	PVA (mL) (8% *w/v*)	AA (g)	BMA (g)	YE (0.8% *w/v*) (mL)
2	25	0.84	0.25	0.7
